# Assessment of Knowledge of Diabetes Mellitus in the Urban Areas of Klang District, Malaysia

**DOI:** 10.3390/pharmacy5010011

**Published:** 2017-02-23

**Authors:** Sasikala Chinnappan, Palanisamy Sivanandy, Rajenthina Sagaran, Nagashekhara Molugulu

**Affiliations:** 1 Department of Life Sciences, International Medical University, Kuala Lumpur 57000, Malaysia; SasikalaChinnappan@imu.edu.my; 2 Department of Pharmacy Practice, International Medical University, Kuala Lumpur 57000, Malaysia; 3 School of Pharmacy, La Trobe University, Bendigo 3552, Australia; thina_1515@hotmail.com; 4 Department of Pharmaceutical Technology, International Medical University, Kuala Lumpur 57000, Malaysia; NagashekharaMolugulu@imu.edu.my

**Keywords:** diabetes mellitus, knowledge, cardiovascular disease, amputations

## Abstract

Diabetes is the most common cause of non-traumatic lower limb amputations and cardiovascular diseases. However, only a negligible percentage of the patients and subjects knew that the feet are affected in diabetes and diabetes affects the heart. Hence, a cross-sectional study was carried out to evaluate the knowledge of diabetes mellitus among the public of different age group, gender, ethnicity, and education level. A sample of 400 participants was randomly selected and data was collected using a structured questionnaire under non-contrived setting. The results showed that there is a statistically significant difference in knowledge on diabetes mellitus among different age groups and different ethnic origin but there is no significant difference in the knowledge among different gender and education level. Out of 400 respondents, 284 respondents (71%) knew that diabetes mellitus is actually a condition characterized by raised blood sugar. Age and education level of respondents were found to be the predominant predictive factors on diabetes knowledge, whereas the gender of respondents did not affect the findings of this study. An improved and well-structured educational programme that tackles the areas of weaknesses should be recommended to increase the level of knowledge on diabetes among Malaysians.

## 1. Introduction

Diabetes mellitus is a group of metabolic diseases whose common feature is an increase in the blood glucose level [1]. It is one of the most common diseases, causing significant mortality and morbidity worldwide. The development of complications in diabetes is not related to hyperglycaemia alone. Studies suggest that the genesis of complications is related not only to glycaemic control but also to blood pressure and lipid control [2].

It is a disease with serious complications that has now reached epidemic proportions and the prevalence rates are expected to go even higher in the future [3]. If the current trend continues, more than 170 million people worldwide will have this disease and this burden is projected to more than double by the year 2030. In Malaysia, diabetes mellitus is a very big growing concern. Significant changes in the lifestyles of Malaysians have contributed to the increased incidence of diabetes. Malaysia, a multiethnic nation consisting of three major races, Malays, Chinese and Indians with a population of about 30 million includes 0.86 million in the Klang district, which has a high epidemic of diabetes mellitus. The World Health Organization (WHO) has estimated that in 2030, Malaysia would have a total number of 2.48 million diabetics compared to 0.94 million in 2000 which is a 164% increase [3,4]. This rising trend is mainly due to some factors such as growing population, aging, urbanization and increasing prevalence of obesity and physical inactivity among Malaysians [5].

Diabetes Mellitus is also associated with long-term consequences that include severe complications. Knowledge is essential for adequate diabetes management and self-management education is the cornerstone of treatment for all people with diabetes. Patients need the knowledge and skills to make informed choices and to facilitate self-directed changes in behaviour and ultimately to reduce the risk of the associated complications [6]. Behaviour and lifestyle changes are the keys to successful self-management of diabetes [7]. Several studies report that the knowledge of diabetes is poor in developing and under-developed countries [8,9,10], and the knowledge should be improved through continuous education by health care professionals like pharmacists, nurses and physicians. Knowledge of diseases are of utmost importance to meet the challenge of increasing healthcare costs. Language is one of the main barriers in Malaysia as the majority of the population are Malay and are mostly educated in Malay language. Even though the literacy rate is higher in Malaysia compared to neighbouring countries in this region, language seems to be a barrier for getting adequate knowledge on any disease [11,12].

Having considered the above factors, it is evident that there is a need to study the knowledge of diabetes mellitus among Malaysians. Hence, in this study an attempt was made to evaluate the knowledge of diabetes mellitus among the public of different age groups, gender, ethnicity, and education level. A pretested and predesigned questionnaire was used to analyse the knowledge of diabetes and the questionnaire was revalidated by the faculties and experts in our institution.

## 2. Materials and Methods

A cross-sectional community-based study was conducted for a period of 4 months. Taman Sri Andalas, Taman Klang Jaya, Bandar Bukit Tinggi and Bandar Botanic of Klang district, Selangor were the study areas for this study. Regardless of ethnicity, a total of 400 participants aged 12 and above were randomly selected by using convenient sampling technique and were asked to participate in this study. Subjects of both genders with no hearing or visual impairment were included. Individuals with significant cognitive impairment and/or psychiatric comorbidity were excluded from the study.

A structured validated questionnaire prepared in American English language was employed to analyse the knowledge on diabetes [13]. The questionnaire consists of socio demographic characteristics of the respondents and an 11 items related to general awareness, symptoms, complications, prevention and control on diabetes mellitus. A study information sheet and written consent form were also included in the survey instrument.

A pilot study was carried out to find out the reliability and validity of the questionnaire. The structured questionnaire was pretested on a sample of 50 Malaysians to find out difficulties in understanding the meaning of the questions and to estimate the amount of time to answer all the questions. The feedback revealed that the questionnaires were easy to understand and quite convenient for the public as they just need to tick the correct options.

Data was collected using convenient sampling methods among the residents of Taman Sri Andalas, Taman Klang Jaya, Bandar Bukit Tinggi and Bandar Botanic. Before data collection, each participant was given a full explanation of the research project and its purpose and was then given to sign an informed consent form. A face-to-face interview was carried out for data collection. Each interview took approximately 10–15 min and was conducted at places comfortable for the participant. When it was necessary, appropriate probing questions were asked. To draw out more complete ideas from the participants, they were given freedom to express additional views on the topic at the end of the interview session.

The collected data were analysed using SPSS version 21.0. Descriptive studies were used to analyse the demographic data obtained whereas Student’s *t* test and one-way analysis of variance were applied as appropriate. The Student’s *t* test was used to compare two groups and one-way analysis of variance (ANOVA) test was used to compare between more than two groups. This study was approved by Ethics committee of AMU (ID: 01BP200904-00068/12/12/), Malaysia. All participants signed the informed consent form.

## 3. Results

In this study, the majority of respondents (49%, *n =* 196) lay within the range of 12–24 years old and only a few respondents (2%, *n =* 8) lay within the range of 64 years old and above. On the other hand, around 32% (*n =* 130) of the respondents fall between 25–44 years old and 16.5% (*n =* 66) of the respondents fall between 45 and 64 years old. The largest percentage of respondents was Indians (50.75%, *n =* 203) and the smallest percent of respondents were others consisting of the Punjabis and Serani (3%, *n =* 12). The Malays and Chinese were 18.5% (*n =* 74) and 27.75% (*n =* 111) respectively. The largest number of respondents received education from college/university (*n =* 236, 59%) and only 7.25% (*n =* 29) of respondents received education from graduate schools. On the other hand, 25% (*n =* 100) of respondents completed high school and 8.75% (*n =* 35) of respondents completed primary school education. Among the respondents in this study, around 58.5% (*n =* 234) were female and the rest 41.5% (*n =* 166) were male. In our study, there are more females in the age group of 12–24 years (*n =* 117) and 25–44 years (*n =* 84), and more males in the age range of 45–64 years (*n =* 36) and >64 years (*n =* 5). As for race, there were more female respondents in all races than males. For the education level there were more female respondents for all except for primary education level. The detailed demographic data are presented in Table 1 and Table 2.

Eleven different structured questions were used to analyse the respondent’s knowledge on causes, symptoms, associated complications, prevention and treatment of diabetes. The analysis revealed that out of 400 respondents, a majority (394; 98.50%) of the respondents knew about diabetes and heard about diabetes mellitus, among this, 233 were female and 161 were male. Around 67.52% (*n* = 158) female and 56.02% (*n* = 93) male participants said any one of their family members had suffered from diabetes.

In terms of their knowledge on what causes diabetes, the majority (*n* = 181; 45.25%) of the respondents agreed that eating more sugar may lead to diabetes, a similar percentage of males (46.39%) and females (44.44%) agreed with the statement. One hundred and forty one respondents (35.25%) agreed that a lack or defect of insulin may cause diabetes, among the respondents who agreed with the statement more were female (37.61%) than male (31.93%).

A total of 197 (84.19%) females and 129 (77.71%) males answered that middle age and elderly people were most commonly affected by diabetes. However, an equal number of female (7.69%) and male (10.85%) said young adults and middle age groups were commonly affected by diabetes.

The majority (*n* = 356; 89%) of the respondents reported that both sex were commonly affected by diabetes, among this 91% were female and 86.1% were male respondents. In terms of the course of this disease, around 82% of female and 80% of male respondents mentioned that diabetes will be lifelong and can be controlled with treatment.

Two hundred and eighty four (71%) respondents consisting of 170 (72.65%) females and 114 (68.68%) males stated that high blood sugar level was the main characteristics of diabetes. However, 27 (11.53%) females and 28 (16.87%) males believed that high urine sugar was the best characteristics of diabetes. The majority of the female and male respondents agreed frequent urination, hunger and thirst are the most common symptoms of diabetes.

The majority of the respondents (*n* = 245; 61.25%) mentioned that a foot problem was the most common complications of diabetes. However, Heart disease (*n* = 111; 27.75%), Kidney disease (*n* = 153; 38.25%), Eye disease (*n* = 130; 32.50%), and Stroke (*n* = 80; 20%) also reported as common complications of diabetes by the respondents. The majority of the respondents agreed healthy diet (*n* = 358; 89.5%), regular exercise (*n* = 294; 73.5%), weight control (*n* = 236; 59%) and stopping smoking (*n* = 133; 33.25%) were the best measures to prevent diabetes. However, 5 (1.25%) respondents said diabetes cannot be prevented.

In terms of the different methods of treatment for diabetes, the majority of the respondents declared drugs (*n* = 351; 87.75%) are the ideal choice of treatment, followed by Insulin (*n* = 346; 86.50%), Healthy diet (*n* = 303; 75.75%), regular exercise (*n* = 256; 64.00%) and weight control (*n* = 206; 51.50%). The data are depicted in Table 3.

The mean scores for both males and females were almost the same, implying that there was no significance difference in knowledge between the females and males (*p* > 0.991). The data are shown in Table 4. ANOVA results shows that the knowledge of respondents with different age groups and ethnicity have significant difference (*p* < 0.001). The respondents aged 12–24 have the highest mean value of 58.92 with standard deviation of 7.81 and the respondents aged 45–64 have the lowest mean value of 54.21 with standard deviation of 7.04 (*p* < 0.001). As for the ethnicity, the Chinese respondents have the highest mean value of 61.42 with standard deviation of 8.75 and the respondents from the other categories consisting of the Serani’s and Punjabi’s have the lowest mean value of 55.26 with standard deviation of 5.91 with a significant value of *p* < 0.001. The data are presented in Table 5.

## 4. Discussion

The study explored the knowledge of diabetes mellitus among the population of Klang Valley, Malaysia. The results showed that there was a statistically significant difference in the mean knowledge of respondents with different age groups and ethnic origin (*p* = 0.000) but there was no significant difference in the knowledge among the gender and level of education (*p* = 0.057). The current literature evaluating the relationship between age and knowledge on diabetes yielded mixed findings [14,15,16,17]. In this study, there were a statistically significant difference in the mean knowledge of respondents with different age groups and the younger respondents have higher knowledge than the older respondents with higher mean value (μ: 58.92). This result is consistent with a study carried out in Ankara, Turkey that found lower level of knowledge of diabetes in older people (18.6%) [18] and in an another study carried out in Singapore that younger respondents have more exposure such as reading more books and using the Internet as sources of information compared to the older respondents [19]. There is a literature evaluating the relationship between gender and knowledge and it was reported that gender is not a determinant of knowledge on diabetes mellitus [20,21]. In Malaysia, males and females are have equal exposure and rights to be educated. This is one of the reasons that the latest Malaysian National Health Morbidity Survey ІV 2015 showed that the prevalence of diabetes mellitus is higher in females at 18.3% compared to males at 16.7% [22].

Level of education was found to be the predominant predictive factors on knowledge of diabetes mellitus [20]. This was in consistent with a study carried out in southern India that level of education influences knowledge on physical activity which is included in the practice section of this questionnaire [23]. However, in this study there were no significant differences in knowledge among respondents of different education level (*p* = 0.057). This is a sign that everyone has almost the same amount of knowledge on diabetes mellitus regardless of their education level. In this study, the comparison between respondents of different ethnicity showed a significant difference in the knowledge of diabetes mellitus. Chinese showed the highest level of knowledge (μ: 61.42) whereas Indians (μ: 55.81) and other ethnics (μ: 55.26) showed the lowest level of knowledge. However, the explanations regarding ethnicity have not been discussed clearly in most literatures.

The study emphasizes that the learning about risk factors of Diabetes Mellitus and its preventive measures are the first step in prevention, since it will enable the public to make the informed decision of adopting a healthy lifestyle [24,25]. In addition to this, health care practitioners and policy makers need consistent good quality data about the distribution and determinants of Diabetes related health issues among their population [26]. These data are essential to design, frame, implement and evaluate successful interventional programs in Malaysia. The results of the study may not entirely reflect the actual knowledge on diabetes among the population of Klang valley, Malaysia. The questionnaire may not be attempted diligently, unless the respondents are educated and advised by the health care providers. The response biases may exist in this study as the respondents may tend to give more positive answers in order to maintain the high level of knowledge; hence, there might be a slight tendency to fill in more positive or neutral responses. In addition, the sample size may not be large enough to obtain reliable and consistent results.

## 5. Conclusions

This study indicated that there is a difference in knowledge on diabetes mellitus among different age groups and ethnicity, whereas the gender of respondents and education level of respondents do not have significant difference. Hence, there is a need to come out with some strategies to enhance the level of understanding on diabetes mellitus. An improved and well-structured educational programme(s) that tackles the areas of weaknesses is recommended to increase the level of public knowledge on diabetes mellitus. Moreover, promotion of implementing healthy lifestyle along with the information about risk factors, diet, exercise, and screening should be encouraged through health campaigns. This can be started as early as in school. This study has major implications to design future educational programmes to control diabetes mellitus. Apart from educating the public, these programmes can be designed in such a way to train and upgrade the health care professions, mainly the physicians and pharmacists, to produce competent diabetes educators who would be able to educate the public on the control of diabetes mellitus.

## Figures and Tables

**Table 1:** Demographic characteristics of respondents (*N =* 400).

Demographic Characteristics	Number of Respondents (N)	Percentage (%)
**Age in years**		
12–24	196	49.00
25–44	130	32.50
45–64	66	16.50
>64	8	2.00
**Sex**		
Female	234	58.50
Male	166	41.50
**Race**		
Malay	74	18.50
Chinese	111	27.75
Indian	203	50.75
Others (Punjabi, Serani)	12	3.00
**Education Level**		
Primary	35	8.75
High School	100	25.00
College/University	236	59.00
Graduate School	29	7.25

**Table 2:** Gender-wise distribution of respondents (*N* = 400).

Demographic Characteristics	Female (*n =* 234)		Male (*n =* 166)		Total	
	N	%	N	%	N	%
**Age in years**						
12–24	117	50.00	79	47.60	196	49.00
25–44	84	35.90	46	27.70	130	32.50
45–64	30	12.82	36	21.70	66	16.50
>64	3	1.28	5	3.00	8	2.00
**Race**						
Malay	50	21.37	24	14.46	74	18.50
Chinese	67	28.63	44	26.50	111	27.75
Indian	108	46.15	95	57.23	203	50.75
Others(Punjabi, Serani)	9	3.85	3	1.81	12	3.00
**Education Level**						
Primary	17	7.26	18	10.84	35	8.75
High School	54	23.08	46	27.71	100	25.00
College/University	142	60.69	94	56.63	236	59.00
Graduate School	21	8.97	8	4.82	29	7.25

**Table 3:** Assessment of knowledge on diabetes and participants’ response (*N =* 400).

Question (s)	Response	Female (*n =* 234)	Male (*n =* 166)	Total (*n =* 400)
Have you heard about Diabetes Mellitus?	Yes	233 (99.57%)	161 (96.99%)	394 (98.50%)
	No	1 (0.43%)	5 (3.01%)	6 (1.50%)
Do any of your family members or relative have/had diabetes?	Yes	158 (67.52%)	93 (56.02%)	251 (62.75%)
	No	76 (32.48%)	73 (43.98%)	149 (37.25%)
As per your knowledge what causes diabetes?	Contact with another diabetic	7 (3.00%)	7 (4.22%)	14 (3.50%)
	Eating more sugar	104 (44.44%)	77 (46.39%)	181 (45.25%)
	Lack/defect of insulin	88 (37.61%)	53 (31.93%)	141 (35.25%)
	Destiny	6 (2.56%)	10 (6.02%)	16 (4.00%)
	Others (Specify)	11 (4.70%)	6 (3.61%)	17 (4.25%)
	Don’t know	18 (7.69%)	13 (7.83%)	31 (7.75%)
Which age groups are most commonly affected by diabetes?	Children and adolescents	4 (1.71%)	5 (3.01%)	9 (2.25%)
	Young adults and middle aged	18 (7.69%)	18 (10.85%)	36 (9.00%)
	Middle aged and elderly	197 (84.19%)	129 (77.71%)	326 (81.50%)
	Others (specify)	2 (0.85%)	1 (0.60%)	3 (0.75%)
	Don’t know	13 (5.56%)	13 (7.83%)	26 (6.50%)
Which sexes are affected by diabetes?	Males only	5 (2.14%)	6 (3.61%)	11 (2.75%)
	Females only	6 (2.56%)	8 (4.82%)	14 (3.50%)
	Both	213 (91.03%)	143 (86.15%)	356 (89.00%)
	Don’t know	10 (4.27%)	9 (5.42%)	19 (4.75%)
What is the course of this disease?	Cures by itself	8 (3.42%)	4 (2.41%)	12 (3.00%)
	Short, cured with treatment	13 (5.56%)	10 (6.02%)	23 (5.75%)
	Lifelong, controlled with treatment	192 (82.05%)	134 (80.73%)	326 (81.50%)
	Others (specify)	1 (0.43%)	0 (0.00%)	1 (0.25%)
	Don’t know	20 (8.54%)	18 (10.84%)	38 (9.50%)
Which of the following best characterizes the disease condition?	High blood sugar	170 (72.65%)	114 (68.68%)	284 (71.00%)
	High urine sugar	27 (11.53%)	28 (16.87%)	55 (13.75%)
	Low blood sugar	7 (3.0%)	5 (3.01%)	12 (3.00%)
	Low urine sugar	0 (0.0%)	1 (0.60%)	1 (0.25%)
	Don’t know	30 (12.82)	18 (10.84%)	48 (12.00%)
What do you think are the most common symptoms of diabetes mellitus? (multiple responses possible)	Frequent urination	145 (61.96%)	86 (51.81%)	231 (57.75%)
	Frequent hunger	65 (27.78%)	67(40.36%)	132 (33.00%)
	Frequent thirst	108 (46.15%)	73 (43.98%)	181 (45.25%)
	Asymptomatic	10 (4.27%)	3 (1.81%)	13 (3.25%)
	Others (specify)	4 (1.71%)	1 (0.60%)	5 (1.25%)
	Don’t know	30 (12.82%)	43 (25.90%)	73 (18.25%)
What are the common complications resulting from diabetes mellitus? (multiple responses possible)	Heart disease	79 (33.76%)	32 (19.28%)	111 (27.75%)
	Kidney disease	110 (47.01%)	43 (25.90%)	153 (38.25%)
	Eye disease	87 (37.18%)	43 (25.90%)	130 (32.50%)
	Stroke	59 (25.21%)	21 (12.65%)	80 (20.00%)
	Foot problems	150 (64.10%)	95 (57.23%)	245 (61.25%)
	Death	41 (17.52%)	38 (22.89%)	79 (19.75%)
	Others (specify)	5 (2.14%)	0 (0.00%)	5 (1.25%)
	Don’t know	19 (8.12%)	21 (12.65%)	40 (10.00%)
What measures can prevent diabetes? (multiple responses possible)	Healthy diet	214 (91.45%)	144 (86.75%)	358 (89.50%)
	Regular exercise	166 (70.94%)	128 (77.12%)	294 (73.50%)
	Weight control	136 (58.12%)	100 (60.24%)	236 (59.00%)
	Quit smoking	65 (27.78%)	68 (40.96%)	133 (33.25%)
	Others (specify)	8 (3.42%)	1 (0.60%)	9 (2.25%)
	Don’t know	8 (3.42%)	7 (4.22%)	15 (3.75%)
	Cannot prevented	4 (1.71%)	1 (0.60%)	5 (1.25%)
What are the methods of treatment in this disease? (multiple responses possible)	Drugs	209 (89.32%)	142 (85.54%)	351 (87.75%)
	Insulin	205 (87.61%)	141 (84.94%)	346 (86.50%)
	Healthy diet	177 (75.64%)	126 (75.90%)	303 (75.75%)
	Regular exercise	146 (62.39%)	110 (66.26%)	256 (64.00%)
	Weight control	117 (50.00%)	89 (53.61%)	206 (51.50%)
	Quit smoking	37 (15.81%)	38 (22.89%)	75 (18.75%)
	Others (specify)	1 (0.43%)	0 (0.00%)	1 (0.25%)
	Don’t know	10 (4.30%)	17 (20.24%)	27 (6.75%)

**Table 4:** Independent sample test for knowledge with different genders.

Sex	N	Mean	Std. Deviation	‘t’ Value	Sig
**Female**	234	57.7	7.5	0.012	0.991
**Male**	166	57.6	8.1		

**Table 5:** Results of questionnaire separated by age, ethnicity and education level.

	Number	Mean	Std. Deviation	Sig
**Age in years**				
12–24	196	58.92	7.81	0.000
25–44	130	57.64	7.52	
45–64	66	54.21	7.04	
>64	8	55.02	6.91	
Total	400	57.73	7.82	
**Ethnicity (race) of the respondents**				
Malay	74	57.23	6.83	0.000
Chinese	111	61.42	8.75	
Indian	203	55.81	6.94	
Others (Punjabi, Serani)	12	55.26	5.91	
Total	400	57.72	7.82	
**Education Level**				
Primary	35	60.66	6.32	0.057
High School	100	57.32	8.23	
College/University	236	57.63	7.98	
Graduate School	29	55.47	6.06	
Total	400	57.72	7.84	

## References

[1] Sarah W., Gojka R., Anders G., Richard S., Hilary K. (2004). Global prevalence of diabetes: estimates for the year 2000 and projections for 2030. Diabetes Care.

[2] ArulKumaran K.S.G., Palanisamy S., Rajasekaran A. (2010). Development and implementation of patient information leaflets in diabetes mellitus. J. Pharma. Health Serv. Res..

[3] Mafauzy M. (2006). Diabetes mellitus in Malaysia. Med. J. Malays..

[4] Murata G.H., Shah J.H., Adam K.D., Wendel C.S., Bokhari S.U., Solvas P.A., Hoffman R.M., Duckworth W.C. (2003). Factors affecting diabetes knowledge in type 2 diabetic veterans. Diabetologia.

[5] Letchumanan G.R., Wan-Nazaimoon W.M., Wan-Mohamad W.B., Chandran L.R., Tee G.H., Jamaiyah H., Isa M.R., Zanariah H., Fatanah I., Ahmad Faudzi Y. (2010). Prevalence of Diabetes in the Malaysian National Health Morbidity Survey III 2006. Med. J. Malays..

[6] Nehad M.H., Istabraq D.A.A., Jyothi V., Hussain Y., Umar F.U. (2012). Assessment of knowledge and awareness of diabetic and non-diabetic population towards diabetes mellitus in Kaduna, Nigeria. J. Adv. Sci. Res..

[7] Linda H., Melinda M., Joni B., Carla E.C., Paulina D., Laura E., Hanson L., Kent D., Kolb L., McLaughlin S. (2014). National standard for diabetes self-management education and support. Diabetes Care.

[8] Palanisamy S., ArulKumaran K.S.G., Rajasekaran A. (2013). Knowledge assessment in adverse drug reactions and reporting. Arch. Pharm. Pract..

[9] Singh A., Shenoy S., Sandhu J.S. (2016). Prevalence of type 2 diabetes mellitus among urban sikh population of Amritsar. Ind. J. Commun. Med..

[10] Acharya K.G., Shah K.N., Solanki N.D., Rana D.A. (2013). Evaluation of antidiabetic prescriptions, cost and adherence to treatment guidelines: A prospective, cross-sectional study at a tertiary care teaching hospital. J. Basic Clin. Pharm..

[11] Ahmad B., Ramadas A., Quek K.F. (2010). The development and validation of diabetes knowledge questionnaire for the Indigenous population in Malaysia. Med. J. Malays..

[12] Najib Mohd N.M., Dali A.F., Ahmad A., Sulaiman S., Hussin S.N., Mokhtar N. (2014). Knowledge and Attitude on Diabetes Among Public In Kota Bharu Kelantan, Malaysia. Int. J. Edu. Res..

[13] Prianka M., Bhaskar P., Debasis D., Nilanjan S., Rachna M. (2010). Perceptions and practices of type 2 diabetics: A cross-sectional study in a tertiary care hospital in Kolkata. Int. J. Diab. Ctries.

[14] Lemes dos Santos P.F., Dos Santos P.R., Ferrari G.S.L., Fonseca G.A.A., Ferrari C.K.B. (2014). Knowledge of Diabetes Mellitus: Does Gender Make a Difference?. Osong Public Health Res. Perspect..

[15] Sivasankari V., Manivannan E., Priyadarsini S.P. (2013). Drug utilization pattern of anti-diabetic drugs in a rural area of Tamilnadu, South India—A prospective, observational study. Int. J. Pharm. Biol. Sci..

[16] Shah V.N., Kamdar P.K., Shah N. (2009). Assessing the knowledge, attitudes and practice of type 2 diabetes among patients of Saurashtra region, Gujarat. Int. J. Diabetes Dev. Ctries.

[17] Rodrigues F.F.L., Zanetti M.L., Santos M.A. (2009). Knowledge and attitude: important components in diabetes education. Rev. Latino-Am. Enferm..

[18] Denis C., Oya O., Esin O., Aysun I. (2006). Evaluation of awareness of diabetes mellitus and associated factors in four health center areas. Patient Educ. Couns..

[19] Tham K.Y., Ong J.J.Y., Tan D.K.L., How K.Y. (2004). How much do diabetic patients know about diabetes mellitus and its complications?. Ann. Acad. Med. Singap..

[20] Lai S.Y., Yahaya H., Noorizan A.A., Ahmed A., Rozina G. (2007). A comparison of knowledge of diabetes mellitus between patients with diabetes and healthy adults: A survey from north Malaysia. Patient Educ. Couns..

[21] Titaporn P., Shu-Chuen L., Hwee-Lin W. (2009). A Survey of Knowledge on Diabetes in the Central Region of Thailand. Int. Soc. Pharm. Outcomes Res..

[22] National Health and Morbidity Survey 2015. http://www.iku.gov.my/images/IKU/Document/REPORT/nhmsreport2015vol2.pdf.

[23] Murugesan N., Snehalatha C., Shobana R., Roglic G., Ramachandran A. (2007). Awareness about diabetes and its complications in the general and diabetic population in a city in southern India. Diabetes Res. Clin. Pract..

[24] Bowman B., Gregg E., Williams D., Engelgau M., Jack L. (2003). Translating the science of primary, secondary, and tertiary prevention to inform the public health response to diabetes. J. Public Health Manag. Pract..

[25] Glasgow R.E., Wagner E., Kaplan R.M., Vinicor F., Smith L., Norman J. (1999). If diabetes is a public health problem, why not treat it as one? A population based approach to chronic illness. Ann. Behav. Med..

[26] Garfield S., Malozowski S., Chin M., Venkat Narayan K., Glasgow R., Green Hiss R., Krumholz H. (2003). Considerations for diabetes translation research in real-world settings. Diabetes Care.

